# Fostering Green Innovation Adoption through Green Dynamic Capability: The Moderating Role of Environmental Dynamism and Big Data Analytic Capability

**DOI:** 10.3390/ijerph191610336

**Published:** 2022-08-19

**Authors:** Danni Yu, Shen Tao, Abdul Hanan, Tze San Ong, Badar Latif, Mohsin Ali

**Affiliations:** 1 School of Business & Economics, University of Putra Malaysia, Serdang 43400, Malaysia; 2 School of Economics and Management, Shandong Youth University of Political Science, Jinan 250103, China; 3 Faculty of Medicine and Health Sciences, University of Putra Malaysia, Serdang 43400, Malaysia; 4 National College of Business Administration and Economics, Lahore 54000, Pakistan; 5 Department of Business Administration, Daffodil International University, Dhaka 1000, Bangladesh; 6 Department of Commerce, Bahauddin Zakariya University, Multan 40000, Pakistan

**Keywords:** green dynamic capability, environmental dynamism, big data analytics capability, green innovation adoption, Pakistani and Malaysian SMEs

## Abstract

Though the concept of green dynamic capability has been increasingly gaining traction among academics, practitioners, and policymakers, its association with green innovation adoption remains unclear. The present study addresses this gap and aims to provide clarity by distinguishing green innovation adoption in the context of developing countries. Drawing on dynamic capability and stakeholder theory, this research shed light on the significance of green dynamic capability for green innovation adoption. Additionally, this study examines the moderating role of environmental dynamism and big data analytics capability in the link between green dynamic capability and green innovation adoption. Adopting a two-wave research design, the sample for this study contained SMEs from Pakistan and Malaysia. Data was obtained from 220 SMEs (105 from Pakistan, 115 from Malaysia). To test the hypotheses, covariance-based structural equation modelling was performed to analyze causal relationships in the model, by using AMOS 23 software. The results showed that green dynamic capability positively impacts green innovation adoption, but environmental dynamism does not positively moderate between green dynamic capability and green innovation adoption. In addition, big data analytics capability positively moderates between green dynamic capability and green innovation adoption. We believe that this study opens a new avenue in the environmental literature under which green innovation adoption is useful for SMEs.

## 1. Introduction

Green dynamic capability has received considerable attention among researchers and business practitioners alike. A substantial body of literature in multiple business fields reveal the remarkable contributing role of green dynamic capability on various firm performance outcomes, including firm reputation [1], brand image [2], network quality [3], asset growth [4], and profitability [5]. Green dynamic capability has therefore been viewed as a potent basis for securing value for firm stakeholders, such as employees [6], customers [7], communities [8], business partners [9], and shareholders [10]. In contrast to research on the effect of green dynamic capability on downstream factors, corporate-level factors—i.e., the antecedents or upstream factors that drive firms’ green innovation adoption (GIA) belong to an area that largely has not been studied in the literature. The need to address this knowledge gap is imperative, since the traditional environmental literature fails to address such fundamental problems. To address the aforementioned literature gaps, the concept of green dynamic capability appears to be significant. Notably, scholars and practitioners argue that excessive consumption of capabilities and resources is an increasingly significant cause of deforestation and economic stagnation [11]. While sustainability issues, such as climate change, environmental pollution, and the sharp depletion of natural resources create problems for world economies, green dynamic capability has emerged as an effective tool against their debilitating effects [6,12]. Appropriate and effective green dynamic capability not only improves environmental performance but also has enormous potential to reduce sustainability issues like climate change, environmental pollution, and the sharp depletion of natural resources [13].

Despite growing scholarly attention toward the concept of GIA [14] and its relationship with firm performance [15], how it is influenced by green dynamic capability remains unclear in literature. Previous research has proposed a negative relationship between GIA and sustainable organizational capabilities, in the form of green product, green consumption, and firm performance [16]. Other studies have found a positive impact of GIA on financial performance [17], while some have contradictorily suggested that GIA does not lead to financial benefits or firm growth [18]. Some past literature also implies that GIA is prominent when there are external organizational factors, including institutional pressure, governmental policies, environmental regulation, competition, and market forces [19]. Moreover, some previous research did not mention any moderator in the GIA–performance relationship. Adding to the body of knowledge, the present study used environmental dynamism and big data analytics as moderators that strengthen the green dynamic capability–GIA relationship, which has rarely been taken into consideration in the literature. Considering the aforementioned literature gaps, the present study addresses this knowledge gap by highlighting the role of green dynamic capability as an emerging concept and discusses the antecedent (green dynamic capability) and outcome (green innovation adoption), which have rarely been taken into consideration in previous research. Additionally, the moderating role of environmental dynamism and big data analytics capability was examined at the nexus of the green dynamic capability–GIA relation. With the abovementioned literature gaps, the present study addresses the following research questions:

1. Does green dynamic capability influence GIA?
2. Does environmental dynamism strengthen the link between green dynamic capability and GIA?
3. Does big data analytics capability strengthen the link between green dynamic capability and GIA?

The aims of the research are to address existing gaps in knowledge, to devise and to test a solution to an existing problem and manifold solution in three distinguished ways. Firstly, the present study addresses that green dynamic capability could help firms to reduce deforestation and environmental problems (e.g., ozone layer depletion, rapid climate change, severe biodiversity imbalance, and environmental degradation) in order to improve green innovation adoption [20]. Our research focuses on small and medium-sized enterprise in Pakistan and Malaysia. To the best of the authors’ knowledge, this research is the prime investigation to examine the role of green dynamic capability as a predictor for green innovation adoption in a developing country context, such as Pakistan and Malaysia. Green dynamic capability exposes firms’ lack of adequate resources and capabilities, while also laying bare other deficiencies, which allowing the manufacturing firms’ to take action to improve biodiversity, preserve the ecosystem, and achieve sustainable development goals. The adoption of green dynamic capability would lead an organization to a ‘win-win’ situation and cause a positive effects on sustainable development and economic growth [21]. Secondly, this study addresses that environmental dynamism strengthens the relationship between green dynamic capability and green innovation adoption. In fact, environmental dynamism has become a vital point of consensus for all manufacturing businesses to advance green growth and concurrently foster environmental sustainability to avoid the deterioration of harmful externalities of green production processes [22]. According to [23], the world’s most considerable sustainability challenge has relied upon firms’ environmental dynamism, which remains necessary for green innovation adoption. Third, this study addresses that big data analytics capability strengthens the relationship between green dynamic capability and green innovation adoption. Adverse effects of green practices pose a substantial concern for practitioners and researchers to execute big data analytics for sustainable development. Big Data analytic capability is often concerned about sustainable development and identifies potential opportunities to provide information to reduce environmental effects. In particular, it has been continually emphasized that big data analytics must run hand-in-hand with addressing environmental concerns and consider green innovation.

The present study addresses significant contributions in three important ways. First, despite green dynamic capability being identified as one of the fundamental drivers of sustainable development, extant literature lacks a theoretically sound and empirically testable framework that can provide specific insights of green dynamic capability and GIA nexus in order to achieve sustainable development. As “go green” is a fundamental concern among manufacturing firms’ worldwide, green dynamic capability has largely been unknown in the green innovation adoption context. Second, the current study investigates green innovation adoption in the context of developing countries, particularly Pakistani and Malaysian manufacturing SMEs. Pakistani and Malaysian manufacturing SMEs were chosen because manufacturing firms’ management systems effectively deals with the green and environmental agenda. Pakistani and Malaysian SMEs are relevant as they are major contributors to the national GDP growth. These [Pakistani and Malaysian] SMEs are also capable of promoting green innovation practices through climate change mitigation, renewable energy, and other sustainable practices [6,24]. Third, the current study employed environmental dynamism and big data analytic capability as a moderator, which strengthens green dynamic capability and the GIA nexus, which has rarely been considered in the related environmental literature [25,26]. The remaining sections of this paper are as follows. The literature review and hypothesis development are presented in Section 2. Section 3 discusses the research methodology and data collection. Data analysis results from covariance-based structural equation modelling (CB-SEM) by using AMOS 23 software are provided in Section 4. Lastly, Section 5 presents the discussion, and Section 6 describes the conclusion, and implications for managers and practitioners.

## 2. Literature Review and Hypothesis Development

Based on the dynamic capability theory and stakeholder theory perspectives, this research shed light on the nexus between green dynamic capability and GIA to achieve sustainable development in developing countries (Pakistan and Malaysia) via the moderating role of environmental dynamism and big data analytics capability. In the present study, the link between green dynamic capability and GIA is suggested by dynamic capability theory, stating that firm strategic capabilities lead toward competitive advantage and superior performance through innovation. Meanwhile, stakeholder theory suggests that stakeholders’ contributions improve firm activities and move firms away from risk and uncertainty. Stakeholders’ involvement is associated with environmental management and sustainability, such that their involvement can foster green dynamic capability and engender elevated performance. Stakeholder theory also suggests that green dynamic capability is considered a source of knowledge that can make an organization cognizant of their core sustainability domains and help them mitigate environmental issues.

### 2.1. Dynamic Capability Theory

The idea that a firm’s ability is integrated, built, and reconfigured by internal and external competencies to adapt to a fast-moving environment was identified by Teece et al. [27], and Eisenhardt & Martin [28] defined dynamic capabilities as an identifiable and specific business process, while Nelson & Nelson [29] stated that dynamic capabilities are foreseeable communicative patterns through which firms manage resources to achieve their objectives, and Teece et al. [27] highlighted that firm capabilities enable the development of innovation. Therefore, dynamic capabilities are engaged in all firm functions and promote the best utilization of capabilities and resources to achieve excellent performance. Dynamic capability theory elucidates how green resources and capabilities influence organizational behavior and reduce environmental uncertainty. Moreover, this theory stresses the contingency context, wherein a firm’s green dynamic capability are linked with its competitive advantage and environmental sustainability. Green innovation has been acknowledged as a mechanism for improving environmental performance that meets the need for a cleaner and greener business environment. Green innovation is thus recognized as a key factor steering competitive advantage. Theoretically, the dynamic capability theory supports the framework of the current study on green dynamic capability and GIA. This theory suggests that core resources and capabilities lead an organization toward competitive advantages and long-term sustainability. In the present study, green dynamic capability are associated with green innovation of SMEs.

### 2.2. Stakeholder Theory

Stakeholder theory, which explains the linkage between firms and their stakeholders, was employed because it is instrumental in explaining environmental actions, behaviors, and strategies [30]. Moreover, stakeholder theory in green innovation research states that managers/senior officials, as core stakeholders in the decision-making of GIA, ultimately form environmental policies. Additionally, the theory states that stakeholders design organizational patterns after receiving an environmental response [31]. This design pattern guides organizations to successfully implement GIA. Stakeholder theory further indicates that stakeholder satisfaction ensures firm survival and success because stakeholder demands are highly associated with the firm’s needs and organizational concerns. It is thus widely accepted that an organization should focus on its stakeholders’ values. As a result, firms perform strategic decision-making to promote broader organizational objectives and address the expectations and demands of their silent stakeholders, such as regulatory authorities, customers, and competitors. Moreover, stakeholders act as implicit and explicit parts that guide firms to improve environmental management practices and attain green innovation.

### 2.3. Green Innovation Adoption

Green innovation acts as a mechanism related to green products and processes that fosters technological innovations to save energy, prevent pollution, recycle waste and hazardous materials, design green products, and encourage environmental management [32]. Green innovation steers an organization away from environmental damage and the wastage of available resources. It creates business strategies and opportunities for the organization to fulfill stakeholders’ green demands, without harming the environment. Moreover, the environmental benefits are not only directed to the firm but also to the society and environment via the transformation of social norms, cultural values, and institutional mechanisms [33]. Green innovation has a pivotal role in leading disruptive and radical SMEs. This is because green SMEs contribute to society with their green products, processes, and business strategies, besides protecting society from the adverse impacts of pollution and climate change [11]. Additionally, green innovation in SMEs reduces environmental footprints by using green production processes, green resource conservation, and renewable green products. Climate change and societal expectations have driven organizational initiatives towards sustainable development, which encompasses the consideration of environmental effects and dynamic changes (e.g., green products, processes, and resource consumption) to acquire environmental performance [34,35]. Research has found that firms’ sustainability matters (e.g., quality of green and environment-friendly products and green processes) have been incorporated in ecological business operations for better product development and environmental performance [14,36]. Green innovation is considered an environmental management agenda because it stimulates environmental performance [37]. Green innovation deals not only with reducing the environmental impacts of hazardous materials but also with firms’ social performance and financial performance, which can be further utilized for better waste management and cost reduction. Green innovation has been well-associated with sustainable development perspectives for driving environmental performance [38]. The linkage between green innovation and sustainable development explored various provisions for a competitive advantage and proactive environmental intention. Green innovation has been revealed to foster firms’ environmental strategies, such as supplier networking and comprehensive environmental management [39,40], to meet stakeholder demands and institutional pressures. Thus, GIA is a critical resource of the firm, facilitating sustainable development and motivating the firm to meet stakeholders’ expectations.

### 2.4. Green Dynamic Capability

The efficacious integration of green dynamic capability makes it easier for an organization to achieve sustainability goals and fulfil the green demand emanating from its stakeholders, thereby offering a competitive advantage [41]. Green dynamic capability creates new products and processes that change the business environment and identify business opportunities for GIA [42]. The integration of firm resources to develop green dynamic capability facilitates the sustainable practices and green initiatives at the operational and strategic level for successful GIA [22]. Integrating green dynamic capability allows an organization to elicit better sustainable performance, particularly in environmental and social aspects. Green dynamic capability transforms sustainable organizational capabilities into environmental performance, such as eco-designing and eco-efficiency initiatives for new product development. As a result, organizations grow their external knowledge for the betterment of green products and green processes in numerous operational activities. In fact, green dynamic capability are better positioned to rely heavily on external knowledge to drive a sustainable development agenda. Additionally, green dynamic capability recognizes the significance of sustainability concerns for long-term sustainable policies and environmental practices. Hence, green dynamic capability advances environmental activities by augmenting formal and informal networks with up-stream and down-stream partners to drive sustainable development. To a certain extent, it is difficult for an organization to implement green innovation without employing their green dynamic capability [43,44]. Firms without green dynamic capability are unable to achieve value creation and competitive advantage as well. Green dynamic capability works as leverage that can transform green knowledge, green strategy, and green actions into sustainable business operations performance for SMEs [45]. It thus acts as a vital element for GIA to reduce negative environmental impacts and to develop a new environmental orientation that can encourage green products and processes to mitigate environmental issues.

### 2.5. Hypothesis Development

#### 2.5.1. Green Dynamic Capability and GIA

Green dynamic capability fosters organizational willingness to pursue proactive environmental actions and to minimize the diverse effects of advanced technologies. To maximize internal integration toward value creation, an organization must make its strategic and technical interdependencies efficient [35]. Considering the dynamic capability and stakeholder perspectives, green dynamic capability consists of exchanging technical information, integrating policy, and establishing a common objective that facilitates decision-making on environmental impacts [46]. The development of sustainable partnerships can systematically guide firms, foster environmental operations, and aid green product development [47]. Green dynamic capability prevents environmental degradation and reveals innovative approaches to achieve environment-friendly solutions for products and processes that often engender green innovation [48,49,50]. Specifically, such collaboration detects and employs technical knowledge and encourages the use of technologies, resources, and productivity functions to achieve environmental innovation. According to [42], green dynamic capability influences suppliers’ environmental practices by impacting buyers’ environmental decisions. Green dynamic capability must therefore be adopted for environmental sustainability and successful value addition of green innovation. Indeed, evidence has suggested that an organization can foster green innovation and reinforce knowledge through different organizational functions, including operations, strategies, and collaborations [51,52,53,54].

From dynamic capability and stakeholder theory, green dynamic capability is the roadmap for sustainable development, as it suggests the acknowledgement of stakeholder concerns for sustainability. Green dynamic capability indicates strategic decision-making with an environmental vision to achieve green products and processes [16]. Green dynamic capability thus creates sustainability awareness, where firms enhance their resources for green products and processes [55]. Resultantly, organizations reduce their hazards and environmental impacts through pollution elimination, stewardship of products, eco-designing, eco-labelling, environmental management system certification (e.g., ISO 14001), and sustainability reporting. Green dynamic capability also influences firms’ environmental values, leading them to actively participate in sustainable activities, such as environmental resource allocation and green innovation. Green dynamic capability, representing a firm’s social, physical, communicative, and psychological assets, impacts the firm’s growth and value creation [56]. Following the dynamic capability theory, green dynamic capability is a critical capability that demonstrates the environmental orientation of employees’ behavior, action, attitude, skill, experience, commitment, and knowledge [57]. Green dynamic capability encourages human resource management (e.g., recruitment and training) that promotes sustainable development and green initiatives. In addition, green dynamic capability has been found to stimulate societies’ readiness for energy efficiency and environment-friendly technologies [58]. According to [59], green dynamic capability creates green job opportunities to meet demand in education, workplace knowledge, cognitive abilities, interpersonal skills, and training and development for non-green careers. As such, it captures the demand for sustainability and GIA arising in the market [58]. Green dynamic capability trains managers and workers with skills and knowledge to enhance their sustainable entrepreneurship and green product design, while reducing their uncertainty and risk tolerance. Consequently, we hypothesized that:

**H1.**  *Green dynamic capability influences GIA.*

#### 2.5.2. Moderating Role of Big Data Analytics Capability

In today’s flourishing “age of data,” leading organizations with extensive big data analytics capabilities can more easily capture product development directions and gain deeper insights into the technical knowledge that drives innovation in a turbulent environment [60]. Big data analytics capability is described as an organizational capability to examine, analyze, process, and deploy big data resources in order for business growth and added values [61]. Big data analytics capability are categorized into the infrastructure flexibility of big data analytics, management capability of big data analytics, and personal expertise big data analytics [62]. These capabilities empower the organization to advance the infrastructure and successfully manage personal experiences, helping the organization to manage resources along with short-term and long-term strategies [15,62]. Similarly, big data analytics can develop internal processes, streamline operational and organizational activities, create better opportunities, and leverage resources for short-term and long-term business success [61,63]. Hence, in the current study, we argued that big data analytics may moderate the relationship between green dynamic capability and green innovation.

On the one hand, big data analytics capability advances organizational resources by developing, deploying, and reallocating process and strengthening internal process, optimizing operational and organizational activities for better efficiency [64,65]. Specifically, organizations with a high usage of big data analytics capability more efficiently deploys and manages the resources from another organization through cooperation. In contrast, organizations that pay less attention to big data analytics capabilities are less likely to be “identified and dominant organizations” in the market and those organizations are far behind, while creating knowledge as compared to their competitors, thus lagging behind in organizational development [66]. Big data analytics capability is favorable to alleviating business risks, helping to acquire valuable resources, identifying business opportunities, and evaluating short-term and long-term strategies. Specifically, organizations with a higher usage of help to advance credible information sources, and big data analytics capability can help the enterprise to identify various potential business opportunities to support the decision-making process [15]. According to dynamic capability perspective, firm acquired resources to gain competitive advantage with two different processes: resource selection and capability development [67]. Big data analytical capability can genuinely analyze the data and support the organization to nominate the valuable resource and capability, add strategic values, further promote capability building, and achieve sustainable development. Despite the importance of big data analytics capability in structuring capability and resources for sustainable development, research linking green dynamic capability with GIA in terms of big data analytics capability is somehow limited. In this regard, the current study elevates the literature by postulating that big data analytic capability fosters a more positive green dynamic capability, which ultimately leads to higher GIA. Examining the moderating role of big data analytic capability is a significant addition to the current debate, particularly in a developing country’s manufacturing industry, which can often be perceived as deceptive or uncaring about dynamic capability. In line with the above arguments, we formulated the following hypothesis:

**H2.**  *Big data analytics capability strengthens the link between green dynamic capability and GIA.*

#### 2.5.3. Moderating Role of Environmental Dynamism

Environmental dynamism is defined as the unpredictable frequency of external environmental changes. With the increasingly volatile changes worldwide, organizations are exploring business opportunities quickly and tackling threats from competitors and the environment, such as environmental turbulence [68]. These dynamic factors need rapid, sustainable strategies to establish sustainable development [69]. Resultantly, the link between GIA and competitive advantage becomes weaker and even becomes negative with sudden environmental changes. Consequently, addressing environmental dynamism is the key to long-term benefits for the organization, as it guides the organization to obtain, adapt, and implement dynamic environmental changes. Environmental dynamism informs firms on their environmental challenges, which can help the achievement of customer demand, high profit, and environmental sustainability [69,70]. Conversely, if an organization neglects environmental changes, it may incur losses. According to [71], environmental dynamism considers increasing the speed of product changes and addressing customer preferences to sustain environmental operations, and [72] similarly stated that environmental turbulence guides the continuous improvement in product/process to respond to environmental changes. If market demands are linked to environmental performance, fluctuations in demand can affect environmental performance [70]. Hence, the effectiveness of GIA may be doubtful when firms cannot respond to sudden environmental changes. It is thus pivotal to understand the role of environmental dynamism between green dynamic capability and GIA. It was therefore hypothesized that:

**H3.**  *Environmental dynamism strengthen the link between green dynamic capability and GIA.*

### 2.6. Gaps in the Literature

This study focuses on green dynamic capability in order to enhance green innovation adoption. Previous scholars focused sparingly on green dynamic capability and green innovation adoption together to be measured. Green dynamic capability is deemed a vital predictor to foster green innovation adoption. Recently, researchers suggested that management should consider green dynamic capability in their decision-making to enhance green innovation practices [73]. Due to some of the following reasons, prior researchers ignored green dynamic capability association with green innovation adoption. First, dynamic capability theory implements an amnesty mechanism along with a string of relief measures of green dynamic capability to promote sustainable development. Unfortunately, the growing concern with environmental issues has overlooked the concept of green dynamic capability. Consequently, the concept of green dynamic capability, which strengthens a firm’s green innovation adoption, is merely unclear in literature to resolve environmental issues and adversely impacts firms’ green innovation practices [74]. Thus, considering the theoretical shortcomings of the green dynamic capability, the present study drew upon the role of green dynamic capability to test the influence on green innovation adoption. Second, prior research on green dynamic capability has associated with green purchasing, environmental training and development, sustainability strategy, and sustainable monitoring [42] yet, how it influences green innovation is still unclear. Consequently, prior research on the impact of green dynamic capability on SMEs’ green practices, in terms of material safety measures, non-toxic chemicals, harmful materials, and green packaging, is still unknown. Second, prior research on the green dynamic capability of SMEs has been questioned. SMEs have failed to incorporate green dynamic capability due to insufficient environmental infrastructures, such as institutional support, green project funding, and technical and managerial support for green activities [48]. However, green dynamic capability can lead SMEs to garner better environmental performance. It is argued that the linkage between green dynamic capability and GIA can tackle environmental issues [75] by developing strategic solutions for environment-friendly infrastructure and waste management. Third, prior research on green dynamic capability suggests different innovative ideas for sustainable development, such as air emissions, energy conservation, waste management, and water conservation [76]. Consequently, prior research on the impact of green dynamic capability on SMEs’ green practices is still unknown. Fourth, earlier research on environmental dynamism appears to moderate the effect of environmental regulations on firm performance [77]. However, as per our best knowledge, this is the first study that examines the moderation effect of environmental dynamism between green dynamic capability and GIA. Finally, a majority of the studies conducted in developed countries on green dynamic capability and green innovation adoption normally found consistent outcomes for firm performance [48]. Despite this, scant concentration has been paid to GIA in the manufacturing industry in emerging countries, such as Pakistan and Malaysia. Thus, to the author’s best knowledge, the current study is a prime investigation to examine green dynamic capability and green innovation adoption to foster sustainable development.

### 2.7. Research Framework

**Figure 1:**
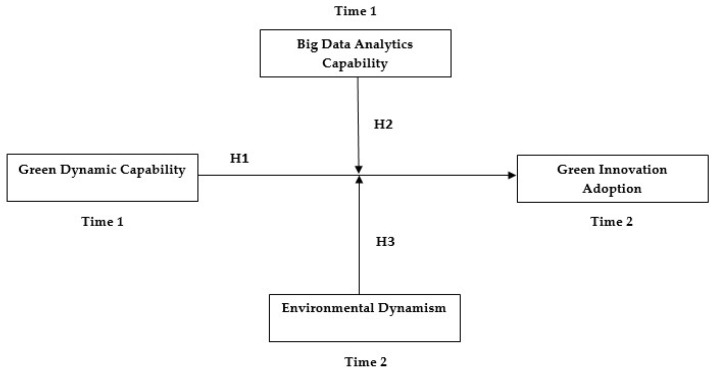
Research framework.

The main objective of this study was to investigate the role of green dynamic capability on green innovation adoption among Pakistani and Malaysian SMEs. Furthermore, it further aimed to ascertain the moderating role of environmental dynamism and big data analytics capability between green dynamic capability and GIA. The research framework is presented in Figure 1.

## 3. Methodology

### 3.1. Sample

The sample of this study comprised Malaysian and Pakistani manufacturing SMEs. The comparative analysis of Pakistani and Malaysian SMEs is noteworthy for a few reasons. Malaysia is considered the most competitive and open economy among developing Asian countries, with a favorable growth outlook [78]. Pakistan is also a developing country that is predicted to welcome positive economic growth, especially in its manufacturing sector [21]. Interestingly, many skilled Pakistani professionals move to Malaysia seeking better jobs and businesses [70]. Both nations’ competitive business environments and workforce transfer call for further investigation of their environmental activities for competitive advantages and long-term survival. Indeed, growing sustainability challenges and business complexity will inevitably entail interest in GIA as a response to environmental problems. In Malaysia, the sample of SMEs was drawn from three major states, namely Selangor, Kuala Lumpur, and Johor, which have the highest contribution to national GDP and the greatest number of SMEs. These states are Malaysia’s most developed and progressive states. These states have well-developed infrastructure for leading industry clusters and are also well-known investment havens with great state governments’ assistance and sophisticated commercial ecosystems. Likewise, in Pakistan, the target sample was drawn from two major provinces with the highest GDP contribution, namely Punjab and Sindh. By using non-probability convenience sampling technique, managers of SMEs in these areas were selected. Based on Cohen’s recommendation, G*Power 3.1.9.2 software was used to investigate the minimum sample size [79]. Based on its calculation with set parameters (f2 = 0.15, α = 0.05, β = 0.20), this research required a sample size of 154.

### 3.2. Two-Wave Research Design

The current study followed a two-wave research design, wherein the measurement of each variable followed a time-based segregation [80]. The independent variables (green dynamic capability) and moderator big data analytic capability were measured at T1, while the moderator (environmental dynamism) and dependent variable (green innovation adoption) were measured at T2. The time lag between T1 and T2 was two to three weeks. This time-based research design addresses potential issues that may arise from self-reported or single-source data. In October 2019, we distributed 500 questionnaires to 105 SMEs in Pakistan of which 271 were completed and returned, yielding a response rate of 54 percent for both time intervals, T1 and T2, respectively. In September 2019, we distributed 700 questionnaires to 115 SMEs in Malaysia, finally receiving 299 completed questionnaires. This produced a response rate of 42.71 percent for both time intervals, T1 and T2, respectively. Table 1 shows the demographic profiles of the SMEs and the individual respondents.

### 3.3. Common Method Bias (CMB)

In the survey method, common method bias identified the potential concern about data, when data collected on different endogenous and exogenous variables with time lag period. Hence, there are some occurrence of CMB. As recommended by Podsakoff, CMB may influence the findings and downgrade the results [80,81]. Therefore, the current study applied Harman’s measured variables to check for potential common bias concern [82]. As for the CMB concern, the highest variance explained by single-factor values must be below 50%. As mentioned in Table 2 Common Method Bias Test, the highest variance values were 45.321%, which describes that the current study does not have CMB concerns.

### 3.4. Measures

Our questionnaire adopted scales from previous research, as mentioned in Table 3 Survey Items. Nine items of green dynamic capability adopted from [47,51,52], which measured the firm’s capability to assesses the track record of the firm regarding specified objectives of environmental quality and resource usage efficiency. Eight items of big data analytics capability were adopted from [83,84], which evaluated the firm’s capability to examine, analyze, process, and deploy big data resources, in order for business growth and added values. Four items of environmental dynamism were adopted from [68], which represents the rate of change in an environment over a time period. Four items of GIA were adopted from [58], which measured the firm’s capacity to introduce technologies for pollution, waste, recycling, and toxic materials to achieve eco-friendly outcomes and to reduce environmental impacts. All items were rated on a five-point Likert scale, where 1 indicated “strongly disagree” and 5 indicated “strongly agree”. The respondents’ and firms’ demographic information was also solicited in Table 1 Demographic Profile.

## 4. Results

### 4.1. Reliability and Validity

To investigate the factor loading of the measurement items, we used exploratory factor analysis (EFA). In addition, Cronbach’s alpha value was used to check the reliability of the model. Based on the factor loading of the confirmatory factor analysis (CFA), we examined the composite reliability. We then proceed to calculate the average variance extracted (AVE) values to determine the convergent validity. In Table 4 Results of measurement model, we described the Pakistan and Malaysian SMEs measurement model results. In Pakistan, AVE valued between (0.854–0.644), composite reliability (CR) for each construct valued between (0.959–0.930), and Cronbach’s alpha valued between (0.946–0.899). In Malaysia, AVE valued between (0.820–0.635), composite reliability (CR) for each construct valued between (0.952–0.923), and Cronbach’s alpha valued between (0.921–0.877). These measurement model values of Pakistani and Malaysian SMEs were above the threshold, reflecting that the constructs have confirmed sufficient criteria and have high reliability and convergent validity. In addition, we further analyzed the goodness-of-fit tests of the five-factor model to synthesize the four latent variables. In Table 5 Mean, standard deviation and correlation, we shows the means, Pearson’s correlation, and standard deviation (SD).

### 4.2. Hierarchical Regression Analysis

The results of the regression analysis are shown in Table 6 and Table 7, where we tested the moderation effect. Firstly, to eliminate the possible effect of multicollinearity, we centered the main variables on the mean before analyzing the interactive effects. Secondly, the variance inflation factor results for all variables were found below 10.0, indicating that multicollinearity was not such a serious problem in the present study. The results of the hierarchical regression analysis for Pakistan and Malaysia are presented in Table 6 and Table 7. As shown in Table 6 Pakistani SMEs Hierarchical Regression Analysis Results, there is a significant positive relationship between green dynamic capability and GIA (β =0.860, *p* < 0.001 Model 2). In Table 7, there is a significant positive relationship between green dynamic capability and GIA (β = 0.853, *p* < 0.001 Model 2). Hence, H1 was supported. In Table 6, the interaction effects of big data analytics capability between green dynamic capability and GIA found a positively moderating effect (β = 0.252, *p* < 0.001 Model). In Table 7 Malaysian SMEs Hierarchical Regression Analysis Results, the interaction effects of big data analytics capability between green dynamic capability and GIA found a positively moderating effect (β = 0.247, *p* < 0.001 Model). Hence, its recommendation of H2 was supported. Lastly, in Table 6, the interaction effects of environmental dynamism exhibited a negatively moderating effect on the green dynamic capability–GIA relationship (β = −0.166, *p* < 0.05, Model 4). In Table 7, interaction effects of environmental dynamism exhibited a negatively moderating effect on the green dynamic capability–GIA relationship (β = −0.161, *p* < 0.05, Model 4). Hence, its confirmation of H3 was not supported.

## 5. Discussion and Implications

### 5.1. Discussion

Our study supports findings that green dynamic capability is an effective and direct way to advance green innovation adoption [35,46] for sustainable development, particularly in a developing country context (e.g., Pakistan and Malaysia). Green dynamic capability showcases the potential to advance green innovation adoption and is most effective when accompanied by reinforcing measures. At the same time, we shed light on the often-neglected role of environmental dynamism and big data analytics capability, which strengthens the green dynamic capability and green innovation adoption link. Drawing on dynamic capability theory and stakeholder theory, the present study sets out to address three important research questions: Does green dynamic capability influence GIA? Does environmental dynamism moderate the relationship between green dynamic capability and GIA? Does BDA capability moderate the relationship between green dynamic capability and GIA? For the first objective, H1 describes the positive impact of green dynamic capability on GIA. H1 was significant in Pakistan and Malaysia, and these results are consistent with the past literature [35]. The results of H1 highlights that green dynamic capability has a positive relationship with GIA. This result is consistent from past empirical studies that indicated a positive relationship [47,51]. Consistent with these findings, our first objective emphasizes that green dynamic capability acts as a strong predictor of GIA in Pakistani and Malaysian SMEs. SMEs are thus more reliant on dynamic capabilities to drive green innovation, on account of their flexible management and lower bureaucracy structures. In Pakistani and Malaysian SMEs, managers recognize the prominence of sustainability concerns for their long term-sustainable development, and they have identified the importance of sustainable polices for their stakeholders and human resource concerns.

The second objective addresses the moderating role of environmental dynamism between green dynamic capability and GIA, which was found to be insignificant in both Pakistan and Malaysia. These results contradict the past literature [85,86,87]. Previous research has even reinforced the positive role of environmental dynamism in shaping GIA and innovation performance [77,88]. While this research does not concur with previous research findings, businesses should still manage their environmental dynamism to achieve a competitive advantage. In fact, Pakistani and Malaysian SMEs are still in their infancy, with many unable to sustain themselves without continuous support from stakeholders (e.g., government, customers, suppliers, etc.). These firms face further challenges from environmental dynamism, including increasing environmental pressures, surging energy issues, raw material scarcity, and mounting pollution. Additionally, manufacturing firms have accustomed their business plans and actions toward competitiveness, setting aside environmental issues. In Pakistan and Malaysia, environmental dynamism is crucial for the establishment of green innovation and the three pillars of sustainability, i.e., environmental, social, and economic. As such, SMEs need to understand resource allocations and to adopt the necessary changes in their operational activities as well as work systems to maintain a sustainable vision. Findings suggest that, managers have the responsibility and authority to pursue change management strategies for a sustainable environment and better environmental transition in SMEs.

The third objective describes the moderating role of BDA capability between green dynamic capability and GIA. The results of H3 highlighting the positively moderating role of BDA capability between green dynamic capability and GIA. These results are consistent with the past literature [25,89]. Findings revealed that big data analytics capability is favorable for alleviating business risks, helping to acquire valuable resources, identifying business opportunities, and evaluating short-term and long-term strategies for achieving sustainable development. Specifically, organizations with higher usage of big data analytics capability help to advance credible information sources, and big data analytics capability can also help companies identify potential business opportunities and support decision-making [15].

### 5.2. Theoretical Implication

This research has several implications for literature and theory. First, this research contributes to dynamic capability theory [27,90] and stakeholder theory [91]. Specifically, we have proven that green dynamic capability shapes and promotes sustainable development within organizations, which in turn exerts influence on GIA. Applying the dynamic capability theory to GIA, this research suggests that green dynamic capability are critical resources that create value for organizations competing in the dynamic market. Similarly, stakeholder theory supports that green dynamic capability does not just drive GIA but also plays a monitoring role for the SDGs agenda. Second, previous research has emphasized the mediating effect of green dynamic capability between green innovation antecedents and outcomes. However, green dynamic capability as an antecedent to green innovation research has thus far been underexplored and unclear. Instead, previous research has confirmed the positive associations between corporate environmental consciousness and green innovation, managerial environmental concern and green innovation, and green patenting and green innovation. Our finding adds value by suggesting that green dynamic capability is indeed an antecedent of sustainable development for enhancing GIA. Third, this research extends the literature of cleaner production by successfully linking green dynamic capability and GIA to promote sustainable development in the SME context.

### 5.3. Managerial Implication

Our research carries several implications for managers. First, we suggest that sustainable organizational capabilities create benefits for the firm as they capture the attention of stakeholders and project a good image, especially given that firms face immense pressure to promote green practices in their business operations. Our results inform public policy makers on the importance of GIA for environmental regulatory legislation as well. Moreover, GIA fulfills the demands of institutional pressure from external stakeholders for green management, and drives environmental performance in business operations. Since SMEs are vulnerable to environmental dynamism problems, incorporating green management and green innovation may promote their adaptability to environmental dynamism. In addition, the moderating role of environmental dynamism between green innovation and environmental performance is more sensitive to the dynamic environment of the firm, which directly and indirectly advance the new technology. From this perspective, the firm may consider their spending for introducing new technology, which can minimize environmental issues and limit carbon emission levels.

### 5.4. Limitation and Future Research

Our study has several limitations and directions for future research. First, the study was conducted in the manufacturing sector, limiting the generalization of findings to the services and retail sectors. Therefore, we propose that future research extend the current framework to non-manufacturing sectors. Second, this research was conducted in two developing countries (i.e., Pakistan and Malaysia). It would be interesting to compare our results with those of developed countries (e.g., the European Union). Third, this research failed to establish environmental dynamism and BDA capability as moderators of the green dynamic capability-GIA link. Future research should address other internal and external factors (e.g., environmental strategy, stakeholder pressure) that moderate this link for a better understanding of SMEs’ green innovation and environmental performance.

## 6. Conclusions

The growing scholarly trend has given a tremendous boost to SMEs’ sustainable development through strategic efforts, such as green dynamic capability. The implementation of green dynamic capability may illuminate critical strategies for firms to achieve a competitive advantage and enhance green innovation adoption. Based on dynamic capability theory and stakeholder theory, this research highlights the pivotal role of green dynamic capability on GIA of SMEs in two developing countries (i.e., Pakistan and Malaysia). A burgeoning track of literature has started to explore green innovation adoption in SMEs operating in developing and under-developed economies, such as Malaysia and Pakistan, due to the overwhelming acknowledgement of the positive role green innovation adoption plays in the developing world. To the best of our knowledge, this is an early attempt to explore the green dynamic capability-GIA link in the context of developing countries. We found that green dynamic capability positively influenced GIA, which in turn promoted sustainable development. However, the moderating role of environmental dynamism between green dynamic capability and GIA was not significant. In addition, the moderating role of BDA capability between green dynamic capability and GIA was significant. Green dynamic capability has thus emerged as a powerful determinant of GIA, which should be given serious consideration by SMEs in developing countries. The results revealed that the concept of green dynamic capability is still in its embryonic stages in these developing nations, despite the growing scholarly attention it has achieved in other fields. Therefore, the present study is the first to determine whether extant measures and constructs are generalizable across multiple developing nations.

## Figures and Tables

**Table 1:** Demographic Profile.

Total Sample	Category	Malaysia (299)		Pakistan (271)	
		Frequency	(%)	Frequency	(%)
**Gender**	Male	109	36.5	234	86.3
	Female	190	63.5	37	13.7
**Firm size**	1–500	43	14.4	38	14.0
	501–500	70	23.4	49	18.1
	1001–1500	89	29.8	74	27.3
	Above 1500	97	32.4	110	40.6
**Ownership Structure**	State Owned and Collective firms	150	50.2	103	38.0
	Private firms	64	21.4	87	32.1
	Foreign invested firms	85	28.4	81	29.9
**Industry Type**	Chemical and Pesticide	81	27.1	94	34.7
	Fertilizer	64	21.4	70	25.8
	Textile	79	26.4	62	22.9
	Food and Beverage	75	25.1	45	16.6
**Firm Age (Years)**	1–10	121	40.5	80	29.5
	11–20	102	34.1	66	24.4
	21–30	44	14.7	74	27.3
	Above 30	32	10.7	51	18.8
**Total**		**299**	**100.0**	**271**	**100.0**

**Table 2:** Common Method Bias Test.

	Initial Eigenvalues Values Components			Extraction Sums of Squared Loadings		
	Total	% of Variance	Cumulative %	Total	% of Variance	Cumulative %
1	15.830	45.321	45.321	15.830	45.321	45.321
2	2.374	9.498	72.819	2.374	9.498	72.819
3	0.818	3.274	76.093	0.818	3.274	76.093
4	0.577	2.308	78.401	0.577	2.308	78.401
5	0.536	2.145	80.545	0.536	2.145	80.545
6	0.513	2.050	82.595	0.513	2.050	82.595
7	0.499	1.997	84.593	0.499	1.997	84.593
8	0.413	1.652	86.245	0.413	1.652	86.245
9	0.400	1.601	87.846	0.400	1.601	87.846
10	0.370	1.480	89.326	0.370	1.480	89.326
11	0.334	1.336	90.662	0.334	1.336	90.662
12	0.316	1.264	91.926	0.316	1.264	91.926
13	0.295	1.179	93.104	0.295	1.179	93.104
14	0.290	1.160	94.265	0.290	1.160	94.265
15	0.267	1.067	95.332	0.267	1.067	95.332
16	0.241	0.963	96.295	0.241	0.963	96.295
17	0.226	0.905	97.201	0.226	0.905	97.201
18	0.205	0.821	98.021	0.205	0.821	98.021
19	0.167	0.667	98.688	0.167	0.667	98.688
20	0.150	0.601	99.289	0.150	0.601	99.289
21	0.080	0.322	99.611	0.080	0.322	99.611
22	0.063	0.252	99.863	0.063	0.252	99.863
23	0.020	0.080	99.943	0.020	0.080	99.943
24	0.011	0.044	99.987	0.011	0.044	99.987
25	0.003	0.013	100.000	0.003	0.013	100.000

**Table 3:** Survey Items.

Green Dynamic Capability [47,51,52]
Our firm has the ability and can quickly monitor the environment to identify new green opportunities.
Our firm has effective routines to identify and develop new green knowledge.
Our firm has the ability to develop green technology.
Our firm has the ability to assimilate, learn, generate, combine, share, transform, and apply new green knowledge.
Our firm has the ability to successfully integrate and manage specialized green knowledge within the company.
Our firm has the ability to successfully coordinate employees to develop green technology.
Our firm has the ability to successfully allocate resources to promote green initiatives.
Our firm has the ability to successfully participate in decision making to promote green initiatives.
Our firm has the ability to successfully participate for using temporary task forces to coordinate green activities.
Big Data Analytics Capability [83,84]
Our firm has excellent expertise to process structural data.
Our firm has excellent analytics personnel (i.e., team) and actively get insights from unstructured data.
Our firm effectively process complicated data and information.
Our firm has programming skills of our personnel that greatly help us to get analytical insights from the large datasets.
Our firm has personnel effectively to get insights from web-based data.
Our firm has effectively use real-time information for day-to-day operations.
Our firm has IT infrastructure strongly focused on information integration by using advanced technology.
Our firm frequently disseminates useful information across our departments.
Environmental Dynamism [68]
Our firm adopts major changes in the modes of production and services provision.
Our firm adopts a high rate of innovation.
Our firm adopts major changes in consumer demographics.
Our firm adopts frequent and major changes in government regulations.
Green Innovation Adoption [58]
Our firm adopts fewer inputs to minimize environmental risks.
Our firm adopts cleaner technologies.
Our firm reusse or recycles inputs, materials, and wastes.
Our firm cannot substitute toxic materials with eco-friendly one.

**Table 4:** Results of measurement model.

Constructs	Items	Standardized Factor Loading (λ)		Cronbach’s Alpha (α)		Composite Reliability		AVE	
		MYS	PAK	MYS	PAK	MYS	PAK	MYS	PAK
Green Dynamic Capability	GDC1	0.876 ***	0.874 ***	0.917	0.930	0.940	0.942	0.635	0.644
	GDC2	0.822 ***	0.820 ***						
	GDC3	0.769 ***	0.784 ***						
	GDC4	0.801 ***	0.801 ***						
	GDC5	0.811 ***	0.805 ***						
	GDC6	0.783 ***	0.791 ***						
	GDC7	0.801 ***	0.830 ***						
	GDC8	0.761 ***	0.764 ***						
	GDC9	0.742 ***	0.748 ***						
Big Data Analytics Capability	BDAC1	0.802 ***	0.812 ***	0.919	0.946	0.952	0.959	0.714	0.746
	BDAC2	0.816 ***	0.846 ***						
	BDAC3	0.853 ***	0.850 ***						
	BDAC4	0.763 ***	0.773 ***						
	BDAC5	0.858 ***	0.888 ***						
	BDAC6	0.917 ***	0.947 ***						
	BDAC7	0.847 ***	0.837 ***						
	BDAC8	0.893 ***	0.943 ***						
Environmental Dynamism	ED1	0.901 ***	0.971 ***	0.921	0.942	0.948	0.954	0.820	0.854
	ED2	0.911 ***	0.941 ***						
	ED3	0.898 ***	0.878 ***						
	ED4	0.911 ***	0.903 ***						
Green Innovation Adoption	GIA1	0.850 ***	0.860 ***	0.877	0.899	0.923	0.930	0.750	0.768
	GIA2	0.861 ***	0.868 ***						
	GIA3	0.872 ***	0.882 ***						
	GIA4	0.881 ***	0.895 ***						

Abbreviations: GDC, Green Dynamic Capability; BDAC, Big Data Analytics Capability; ED, Environmental Dynamism; GI, Green Innovation; AVE, average variance extracted. Significant at *** *p* < 0.001.

**Table 5:** Mean, standard deviation and correlation.

	Constructs (Pakistan)	Mean (SD)	1	2	3	4	5	6	7	8	9
1	Gender	1.07 (0.27)	1								
2	Firm Size	2.73 (0.85)	0.016	1							
3	Firm Age	1.68 (0.78)	−0.004	−0.093	1						
4	Industry Types	1.95 (0.89)	0.125	−0.125	−0.107	1					
5	Ownership Structure	1.77 (0.90)	−0.069	0.126	−0.058	−0.139	1				
6	Green Dynamic Capability	4.36 (0.68)	0.051	−0.064	0.013	0.026	−0.055	**0.802**			
7	Big Data Analytics Capability	4.31 (0.70)	0.064	−0.077	0.045	0.030	−0.026	0.782	**0.864**		
8	Environmental Dynamism	4.24 (0.84)	0.076	−0.109	0.060	0.007	−0.027	0.703	0.834	**0.924**	
9	Green Innovation Adoption	4.21 (0.83)	0.074	−0.144	0.024	0.069	−0.067	0.716	0.621	0.726	**0.876**
	**Constructs (Malaysia)**	**Mean (SD)**	**1**	**2**	**3**	**4**	**5**	**6**	**7**	**8**	**9**
1	Gender	1.03 (0.21)	1								
2	Firm Size	2.69 (0.79)	0.014	1							
3	Firm age	1.63 (0.73)	−0.003	−0.089	1						
4	Industry types	1.91 (0.83)	0.121	−0.123	−0.102	1					
5	Ownership Structure	1.67 (0.87)	−0.063	0.121	−0.051	−0.131	1				
6	Green Dynamic Capability	4.29 (0.65)	0.047	−0.059	0.017	0.029	−0.051	0.797			
7	Big Data Analytics Capability	4.26 (0.63)	0.059	−0.071	0.048	0.021	−0.028	0.771	0.845		
8	Environmental Dynamism	4.19 (0.76)	0.071	−0.101	0.047	0.012	−0.022	0.689	0.819	0.906	
9	Green Innovation Adoption	4.15 (0.77)	0.068	−0.139	0.019	0.063	−0.062	0.697	0.604	0.717	0.866

**Table 6:** Pakistani SMEs Hierarchical Regression Analysis Results.

Variables	Green Innovation			
Model Path	Model 1	Model 2	Model 3	Model 4
Control Variable				
Gender	0.212 (0.300)	0.107 (0.457)	0.047 (0.692)	−0.008 (0.909)
Firm Size	−0.131 * (0.044)	−0.091 * (0.040)	−0.077 * (0.042)	−0.040 (0.088)
Firm Age	0.015 (0.032)	0.010 (0.833)	−0.020 (0.621)	−0.029 (0.259)
Industry Types	0.032 (0.553)	0.028 (0.848)	0.025 (0.431)	0.038 (0.062)
Ownership Structure	−0.036 (0.558)	−0.008 (0.848)	−0.031 (0.384)	−0.024 (0.284)
Independent Variable				
Green Dynamic Capability (GDC)		0.860 *** (0.000)	0.475 *** (0.000)	0.592 *** (0.000)
Moderators				
Big Data Analytics Capability (BDAC)			0.998 *** (0.000)	0.568 *** (0.000)
Environmental Dynamism (ED)			−0.385 *** (0.000)	−0.477 *** (0.000)
Interaction Terms				
GDC × BDAC				0.252 *** (0.000)
GDC × ED				−0.166 * (0.044)
R^2^	0.030	0.525	0.684	0.878
ΔR^2^	0.009	0.512	0.673	0.873
F Value	1.42	41.9 ***	61.2 ***	70.3 ***

Note: * *p* < 0.05; *** *p* < 0.001. Standard errors in parentheses.

**Table 7:** Malaysian SMEs Hierarchical Regression Analysis Results.

Variables	Green Innovation			
Model Path	Model 1	Model 2	Model 3	Model 4
Control Variable				
Gender	0.209 (0.297)	0.103 (0.451)	0.041 (0.689)	−0.005 (0.897)
Firm Size	−0.129 * (0.044)	−0.087 * (0.040)	−0.073 * (0.038)	−0.039 (0.081)
Firm Age	0.012 (0.029)	0.009 (0.829)	−0.018 (0.619)	−0.026 (0.253)
Industry Types	0.029 (0.549)	0.026 (0.841)	0.021 (0.427)	0.033 (0.057)
Ownership Structure	−0.031 (0.552)	−0.003 (0.839)	−0.027 (0.377)	−0.019 (0.279)
Independent Variable				
Green Dynamic Capability (GDC)		0.853 *** (0.000)	0.471 *** (0.000)	0.587 *** (0.000)
Moderators				
Big Data Analytics Capability (BDAC)			0.989 *** (0.000)	0.561 *** (0.000)
Environmental Dynamism (ED)			−0.381 *** (0.000)	−0.469 *** (0.000)
Interaction terms				
GDC × BDAC				0.247 *** (0.000)
GDC × ED				−0.161 * (0.039)
R^2^	0.027	0.519	0.679	0.871
ΔR^2^	0.007	0.509	0.667	0.863
F Value	1.39	40.7 ***	59.3 ***	69.8 ***

Note: * *p* < 0.05; *** *p* < 0.001, Standard errors in parentheses.

## Data Availability

The data presented in this study are available on request.
